# Narrow-Gap Rheometry: A Novel Method for Measuring Cell Mechanics

**DOI:** 10.3390/cells11132010

**Published:** 2022-06-23

**Authors:** Khawaja Muhammad Imran Bashir, Suhyang Lee, Dong Hee Jung, Santanu Kumar Basu, Man-Gi Cho, Andreas Wierschem

**Affiliations:** 1 German Engineering Research and Development Center, LSTME-Busan Branch, Busan 46742, Korea; imran.bashir@lstme.org (K.M.I.B.); suhyang.lee@lstme.org (S.L.); donghee.jung@lstme.org (D.H.J.); mgcho@gdsu.dongseo.ac.kr (M.-G.C.); 2 Institute of Fluid Mechanics, Friedrich-Alexander-University Erlangen-Nürnberg (FAU), 91058 Erlangen, Germany; santanu.k.basu@fau.de; 3 Division of Energy and Bioengineering, Dongseo University, Busan 47011, Korea

**Keywords:** cell monolayer, cell rheology, drug screening, human diseases, mechanobiology, mechanophenotyping

## Abstract

The viscoelastic properties of a cell cytoskeleton contain abundant information about the state of a cell. Cells show a response to a specific environment or an administered drug through changes in their viscoelastic properties. Studies of single cells have shown that chemical agents that interact with the cytoskeleton can alter mechanical cell properties and suppress mitosis. This envisions using rheological measurements as a non-specific tool for drug development, the pharmacological screening of new drug agents, and to optimize dosage. Although there exists a number of sophisticated methods for studying mechanical properties of single cells, studying concentration dependencies is difficult and cumbersome with these methods: large cell-to-cell variations demand high repetition rates to obtain statistically significant data. Furthermore, method-induced changes in the cell mechanics cannot be excluded when working in a nonlinear viscoelastic range. To address these issues, we not only compared narrow-gap rheometry with commonly used single cell techniques, such as atomic force microscopy and microfluidic-based approaches, but we also compared existing cell monolayer studies used to estimate cell mechanical properties. This review provides insight for whether and how narrow-gap rheometer could be used as an efficient drug screening tool, which could further improve our current understanding of the mechanical issues present in the treatment of human diseases.

## 1. Introduction

The mechanical properties of cells have vital functional implications, such as mechanical stability, adjustment to environmental load, motility, proliferation, phagocytosis, contraction, and morphogenesis [1]. Cell mechanics defines response of the cells to mechanical forces exerted by the cell microenvironment, such as the extracellular matrix [2]. Cells, by detecting changes in the extracellular matrix via a process known as mechanosensing [3], modify their responses, such as cytoskeletal organization, adhesion, stiffness, and motility [4]. In eukaryotic cells, the cytoskeleton is responsible for controlling these mechanical properties, cell shape, locomotion, and division. Their mechanical properties are governed by the actin–myosin, the microtubule, and the intermediate filament networks. These cytoskeleton components are connected to each other. The contractile forces built up by myosin motors are counterbalanced by forces transmitted from the adhesive contacts of a cell to its environment via the actin–myosin network. Intermediate filaments contribute significantly to the mechanical response of the cells at large cell deformation when they are stretched [5]. Compared to those, microtubules are stiff [6] and play a vital role in mitosis [7].

The mechanical cell properties are useful and promise label-free markers of cell state, indicating cytoskeletal or nuclear changes due to various diseases that change the mechanical cell properties [8]. Malignant transformation and neoplasm are related to significant changes in the cellular cytoskeleton and surface alterations, hence in the mechanical cell properties [9,10]. Many diseases show deviations in the structural and mechanical cell properties. Among them are arthritis, asthma, elliptocytosis, malaria, sickle cell anemia, spherocytosis, osteoarthritis, inflammation, and, particularly, cancer [11,12], which is the second leading cause of death worldwide [10]. The changes in the cytoskeleton are crucial in the pathology of diseases like cancer where growth, spreading, and metastasis are facilitated by altered cell mechanics [13,14,15].

The decrease in cell stiffness, due to changes in internal and cytoskeletal structures, could predict an aggressive and metastatic state of cancer cells [16,17,18]. Cancer cells are found to be softer at small deformations [8,10,13,14,19,20]. It has been shown that cell softening is a good indicator for increased cell proliferation and may help detect early dysplasia, for instance, in oral cancer [14]. Hence, cell-rheological measurements are envisioned to serve as a tool for personalized medicine [8]. The cancer cell characteristics are highly affected during the epithelial–mesenchymal transition, resulting in altered cell–cell and cell–matrix interactions, motility, and invasiveness [21,22]. Invasiveness, on the other hand, is believed to be related to the ability of cells to generate traction forces [23]. Differentiating healthy cells from cancerous ones and between various states of cancer by their mechanical properties is expected to be of great medical benefit [10]. A better understanding of mechanical changes during cancer progression is expected to result in novel drugs and treatments, hindering neoplasm propagation by altering mechanical properties of cancerous cells [14].

## 2. Single Cell Studies vs. Average Rheological Properties

For the general understanding of cells, significant efforts have been put into studying the mechanical cell behavior. During the last decades, different techniques, like optical and magnetic tweezers, atomic force microscopy, magnetic twisting cytometry, micropipette aspiration, cell poking, particle tracking micro-rheology, optical stretching, and high-throughput microfluidic techniques have been employed to measure the viscoelastic behavior of single cells [2,6,24,25,26,27,28]. Frequency-dependent changes in the viscoelastic properties of single cells have been studied extensively. Over a wide frequency range, cells show a power-law rheology, like soft glassy materials. The exponents typically range between 0.1 and 0.5, being smaller for stiffer cells [6]. Yet, quantitative data on the absolute value of the cell stiffness or the storage and loss moduli can differ depending on the method employed [12]. One reason is that most of the single-cell studies have been conducted in the nonlinear viscoelastic regime, where the response depends in a nonlinear fashion on the mechanical load. For general mechanical material characterization, the linear regime is mandatory to avoid the damage of the material. Yet, it is often difficult, if not impossible, with methods like optical tweezers, micropipettes, or cell poking to reach or resolve the linear viscoelastic regime. Moreover, optical methods, such as optical stretching with high power input may suffer from considerable heating [2,24]. Different rheological techniques measure various aspects of cell mechanics, and the choice of an appropriate method depends on the question being addressed. Common methods for measuring cell mechanics have been briefly compared in Table 1.

In single-cell studies, it is cumbersome to quantify the average viscoelastic properties of adherent cells. Depending for instance on cell identity, life cycle, shape, structure, and level of proteins, cells show large cell-to-cell variations [62]. Stiffness and dynamic moduli may vary by orders of magnitude [2,62]. On the other hand, drugs, aging, or diseases may strongly affect the cytoskeleton and thus the viscoelastic cellular properties [13]. Hence, to quantify their impact, it is important to determine the average viscoelastic cell properties in the linear viscoelastic range. In single-cell studies, experiments with several tens up to several thousand cells per hour have been conducted [29,62,63,64,65]. Although these methods provide quantitative data for single cells, complex chip design and operational control of microfluidic devices, non-standard culture protocols, reduced proliferation rates, and small volumes challenging subsequent analytical chemistry hold back their application in drug screening or in clinical trials [27,59,60]. Moreover, microfluidic devices are usually made from polydimethylsiloxane. Compared to polystyrene or glass, of which cell culture plates are usually made, it is rather compliant [59,66]. Since adherent cells may be affected by the substrate mechanics, it may result in differences to widely established studies with cell culture plates. Averaging a large number of cells (of the order of a million) can be achieved by studying them in a monolayer between rheometer disks [2,56,67]. Dakhil et al. have extended the method to enable the quantitative determination of average linear viscoelastic cell properties in single experimental runs [20]. This permits the quantifying of the impact of diseases and of drugs on the cells in single experimental runs that would otherwise be difficult if not impossible to determine with low-content methodologies.

Atomic force microscopy is a widely used technique in cellular biophysics, allowing high special resolution including the topography of living cells and the fast and highly sensitive measurement of mechanical cell properties [68]. It allows discriminating pathological and physiological conditions of cells by the mechanical fingerprinting of diseased and healthy cells and distinguishing cancer cells at different stages of malignancy [69,70,71,72,73]. In addition, it allows the investigation of changes in adhesiveness, stiffness, and deformability of cells underlying different processes that affect cell phenotype, including cancer cell transformation and metastasis [28,74,75,76,77]. It is commonly used to quantify the mechanical properties of adherent cells at a subcellular resolution [2]. The quantitative information received by atomic force microscopy is based on the indentation of the cell membrane with a probe or tip situated on the border of a cantilever. Indentations with atomic force microscopy probes of a few nanometer apexes may affect inner cell components due to the pressing of the membrane in between the cortex filaments for depths < 100 nm [26]. Furthermore, cell mechanical measurements using atomic force microscopy could show more than tenfold differences depending on the measurement parameters and the probed region of the cells [2].

The cell mechanical properties could function as a biophysical marker of its state and functions, showing its potential for clinical diagnostics. Recently, single-cell mechanophenotyping using microfluidic-based methods has shown comparable results to flow cytometry [27,78,79,80]. Urbanska et al. compared cell mechanical properties using three different microfluidic-based techniques: constriction-based deformability, shear flow deformability cytometry, and extensional flow deformability cytometry [27]. Traditional single cell techniques, such as atomic force microscopy, micropipette aspiration, optical stretching, and parallel-plate rheology, used to quantify cell deformation under exposure of external stresses, suffer from technically demanding and time-consuming procedures, which limit their use beyond specialized laboratories [27]. Contrary to the traditional single cell studies, the recently developed microfluidic-based techniques allow for the robust, high-throughput assessment of the cell deformability under homogeneous and heterogeneous cell populations. Furthermore, due to the ease of handling, these techniques could be used in biological laboratories and clinical settings. However, the current lack of standardization among various microfluidic techniques poses analytical challenges and limits cross-study comparison.

Due to the difficulty of obtaining representative quantitative data of linear viscoelastic cell properties in single cell studies, the impact of drugs on mechanical cell properties are usually studied first by measuring the viscoelastic properties of a single cell and repeating this measurement after treating the cells with drugs. In this way, it has been shown that substances that affect actin or intermediate filaments like cytochalasin D, latrunculin A, 2,3-butanedione monoxime, blebbistatin, or ML-7 [38,62,81] affect the mechanical cell properties. As a tendency, it has been observed that fixation and contractile agents like histamine or serotonin increase cell stiffness, while relaxing agents like isoproterenol, DBcAMP, and ML-7 decrease it [82]. Yet, depending on their mechanism, some relaxing agents like blebbistatin [38] and latrunculin A [81] have a considerable impact on frequency dependence, while others, like cytochalasin D [38] and sodium azide/deoxyglucose [81], do not.

Wu et al. compared the cell mechanical properties of the breast cancer cell line MCF-7, using most widely adopted methods for cell mechanics, including atomic force microscopy, magnetic twisting cytometry, particle tracking micro-rheology, parallel-plate rheometry, cell monolayer rheology, and optical stretching [2]. Among the compared techniques, they observed a variation by at least two orders of magnitude in the average values of the tested moduli. Apart from the general method employed, they showed that the data depends on specificities of the method. For instance, when using atomic force microscopy, there was more than a tenfold difference in the mechanical properties depending on the measurement parameters and the probed region of the cells. The mechanical cell response also depends sensitively on the forcing and the force profile [2] and on the pre-stress [83]. The positive correlation between pre-stress and elastic modulus observed in cell monolayer rheology was also consistent with atomic force microscopy experiments using a sharp conical probe [2]. These results highlight the importance of selecting an adequate technique for the biological question being addressed.

## 3. Narrow-Gap Rheology

Rheometers are versatile machines to characterize the rheology of materials. To this end, they detect the relation between shear stress, τ, deformation, γ, and shear rate, $\dot{\gamma }$, respectively. In parallel-disk rotational rheometers, these quantities are derived for the maximum values within the gap, i.e., at the outer radius, R, of the gap, from the torque, M, and the angular displacements between the disks, θ, respectively, to the angular velocity, Ω, by Equation (1) [84].

(1) $$\begin{array}{ccc} \tau =\frac{2}{\pi }\frac{M}{R^{3}} & \gamma =\theta \frac{R}{H} & \dot{\gamma }=\Omega \frac{R}{H} \end{array}$$

where H is the gap width between the rheometer disks.

Viscoelastic solids are usually characterized by exposing them to oscillatory load. While for a perfectly elastic solid shear deformation and shear stress are in phase, the phase difference, *δ*, between them is π/2 for a perfect liquid. For a viscoelastic material, the phase difference is in-between. The elastic response is quantified by the storage modulus, *G*′, and the viscous one by the loss modulus, *G*″. They are related to the former quantities by Equation (2) [84].

(2) $$\begin{array}{cc} G^{\prime }=\frac{\tau_{0}}{\gamma_{0}}\cos\delta & G^{\prime \prime }=\frac{\tau_{0}}{\gamma_{0}}\sin\delta \end{array}$$

where the index 0 indicates the amplitude. The moduli usually depend on the oscillation frequency.

Rheometers are usually employed to characterize the bulk behavior of the samples working at characteristic sample thickness in the millimeter range. To study cell rheology, the sample size needs to be reduced to that of the cells, i.e., in the range of a few micrometers. Fernández et al. introduced a narrow-gap rheometer that allowed the studying of the rheology of cells in shear oscillation [56]. To this end, cells were adhered in a monolayer between two flat plates via a thin fibronectin coating. They measured mechanical properties of the fibroblast monolayer and observed a significant drop in cell stiffness when using the actin-depolymerizing drug, latrunculin-A. However, as the cell coverage remained unknown, they were unable to quantify the average cell mechanical properties. Dakhil et al. introduced a narrow-gap device that also allows working at narrow gaps in unidirectional shear [85]. With this device, a parallelism of the plates of better than ±1 µm for unidirectional flows and about ±0.4 µm for oscillation can be reached. This is far below the reported effective zero-gap error of commercial rheometers of 25 µm to 70 µm [86], which, apart from being due to disk roughness and residual disk inclination, can be caused by squeeze flow during zeroing the rheometer [85]. The enhanced precision allows the extending of the measurement window to much higher shear rates [87,88] as well as studying the adhesion [89] and load limits [90] of cells in low viscous environments. With their device, Dakhil et al. reached the linear viscoelastic range at small deformations, where the stress amplitude is proportional to the deformation amplitude [20,91]. By determining the cell coverage in the monolayer, i.e., the number of cells in the measurement gap, they were able to quantify the average mechanical cell properties.

**Figure 1:**
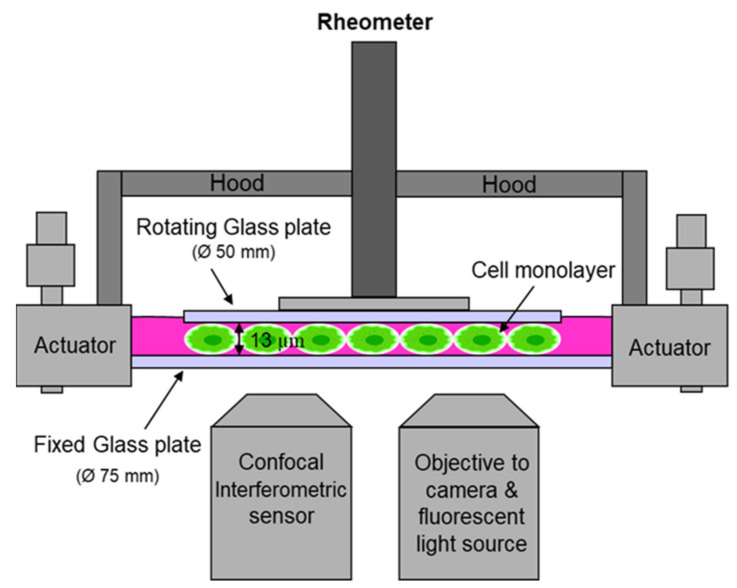
Sketch of the narrow-gap rheometer.

The setup of the narrow-gap device used by Dakhil et al. [85,91] is sketched in Figure 1. The fixed bottom plate has a diameter of 75 mm and an evenness of λ/4. The rotating plate has a diameter of 50 mm with an evenness of λ/4. The top plate is attached to a measurement head of the rheometer. The bottom plate is aligned perpendicularly to the axis of rotation using three actuators. For disk alignment, the gap width is measured with a customized confocal interferometric sensor. It has a working distance of 42 mm, a measuring range of 90 µm and allows the independent detection of the gap width with an axial accuracy of 10 nm. The sensor is placed underneath the fixed glass plate. It is placed next to a fluorescence microscope equipped with a 5x objective, which is used for detecting cell coverage and cell viability. They determined the average area covered by the cells from multiple images at distinct locations after staining the cells with a fluorescent dye. Since deformation is strongest at maximum distance from the turning axis of the rheometer, and, hence, the rheometer signal is caused by the cells at the outer rim, the images are taken close to the edge of the upper plate. To enable measurements at various locations sensor and microscope are both fixed to a traverse. Sander et al. used a similar setup [57]. Differently to Dakhil et al. [20,91], they used small beads for setting up the gap width. Instead of the upper plate, they used a ring to disregard the cells near the axis of oscillation.

**Figure 2:**
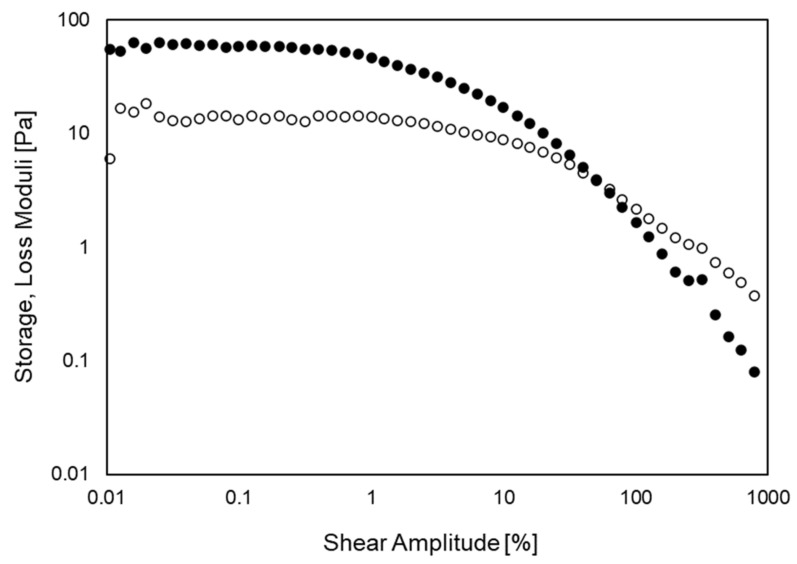
Amplitude sweep of MCF-7 cells treated with paclitaxel. Storage and loss moduli are indicated by closed and open symbols, respectively. Angular frequency: 1 rad/s; Gap width: 13 µm.

The setup of Dakhil et al. [20] enabled the detection of the linear viscoelastic regime of the cells. Figure 2 shows a representative amplitude sweep of MCF-7 cells detected with a setup similar to theirs. In this example, the cells were treated with paclitaxel, a well-known anticancer drug. Storage and loss moduli were determined from the maximum shear-stress and shear-deformation amplitudes, i.e., at the radius of the upper plate. The linear viscoelastic regime appears as a plateau in storage and loss moduli. As is apparent in Figure 2, it is constrained to small amplitudes. With increasing amplitudes, the moduli deviate from the plateau and tend to decline. At larger amplitudes, the cells start to detach from the plates and a cross-over of the moduli appears.

**Figure 3:**
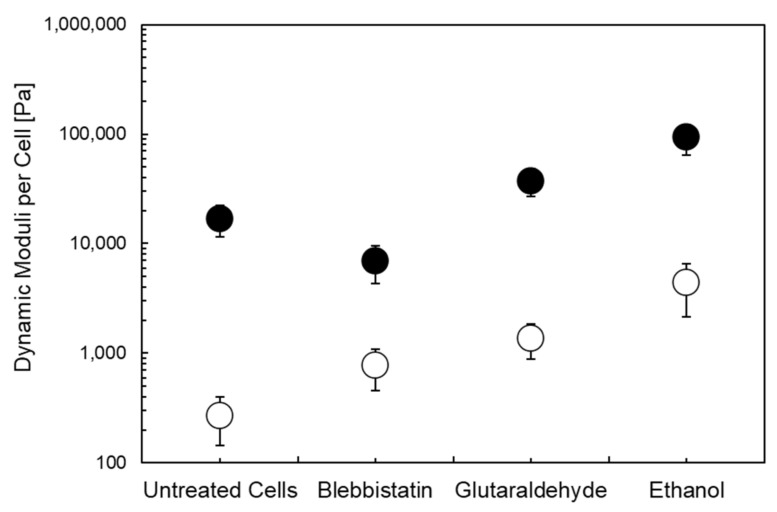
Dynamic moduli per cell for untreated fibroblasts and fibroblasts treated with different drugs [91]. Storage and loss moduli are indicated by close and open symbols, respectively. Frequency: 1 Hz; Gap width: 5 µm.

Biochemical treatments may affect the dynamic moduli and their frequency dependence. To study the impact of drugs that are known to affect the cytoskeleton structure, in a recent study we applied ethanol (0.5 vol. %), glutaraldehyde (0.1 vol. %), and blebbistatin (150 µM) to murine fibroblasts (Figure 3) [91]. Figure 3 shows the average moduli per cell to the linear viscoelastic regime for murine fibroblasts [91]. Compared to untreated cells, blebbistatin lowers the storage modulus while glutaraldehyde and ethanol tend to enhance the storage modulus. Except blebbistatin, all other drugs yielded an increase in the loss modulus. These results correlate with the effects of drug treatment and for setting the experimental limits accordingly.

Recently, Kiran et al. [58] used cell monolayer rheology to study the rheological properties of a serum-starved, fully confluent Madin–Darby canine kidney cell monolayer in shear oscillation with a setup similar to that used by Dakhil et al. [20] and Fernández et al. [20,56]. The circular cell monolayer was grown on rectangular glass coverslips using polydimethylsiloxane strips. Both plates were coated with fibronectin to adhere cells. For plate alignment and optical observation, they used a microscope with a 20x objective.

Elkins et al. introduced a linear cell monolayer rheometer to study the cell mechanics of confluent stromal vascular cells [67]. As an upper plate, they used a slightly curved plano-convex lens with the curves side to the bottom. The curvature let a variation in gap width of less than 5%. They adhered the plate to a mount fixed to a force transducer. To shear the monolayer, the transducer was mounted on a piezoelectric stage and displaced laterally. The device was centered on a metal dish with a glass coverslip mounted onto an inverted microscope with a 100x objective to optically monitor cell density and cell deformation. Both plates were coated with collagen to adhere cells. With this device, Elkins et al. [67] conducted step-strain measurements and detected changes in the average relaxation modulus of the cell monolayer.

The linear monolayer device of Elkins and coworkers differs in a number of ways from that introduced by Fernández et al. [56]: (1) eliminating the need for perfectly parallel top and bottom plates by employing a top plate with gentle curvature; (2) provides high cell spreading area; (3) allows the live quantification of cell strain values during deformation due to the incorporation of live cell imaging system; and (4) uses a linear shear geometry, ensuring in principle a uniform strain exposure of all cells in the system. Similar to other monolayer studies [2,20,56], the linear monolayer device does not require culturing cells on the rheometer plates and show high cell viability. However, their device has the following limitations as compared to the system introduced by Dakhil et al. [20]: (1) does not allow gap width adjustment; (2) requires 24 h for cell attachment; (3) gap width was determined using spacer beads of known diameter, however, this gap width was not well-defined and precise due to the small curvature of the top plate; (4) does not allow changes in normal stresses as the normal force on the cell monolayer was solely due to the weight of the top plate and the pyramid mount.

The rotational devices, as presented by Dakhil et al. [20,91], on the other hand, offer following possibilities: (1) cells can be plated on the rheometer plates a few hours before the experiment thus reducing the time scale of the actual experiment; (2) allows optical determination of cell concentration and live quantification of cell strain values during deformation; (3) allows gap width adjustment with a precision of about ±0.4 μm; (4) allows changes, setting and measuring the normal stresses; (5) allows elongation and compression of cells, hence quantifying the impact of pre-stress on storage and loss modulus; and (6) shows high reproducibility. Together, all these features make the narrow-gap rheometer an attractive choice as a diagnostic tool to quantify variation in cell mechanics, such as in transgenic cell lines. The above-mentioned rheological studies employing narrow-gap rheometer are summed up in Table 2.

## 4. Summary and Future Perspectives

The mechanical characterization of cellular behavior allows a comprehensive understanding of physiological and pathological processes [94,95] with potential applications in clinical diagnostics [8,27,96,97]. Cell mechanical properties define its function and behavior against the mechanical forces exerted by the cell microenvironment or by the presence of external components [98]. This cellular response to the mechanical forces can be elastic, viscous, or viscoelastic as well as active or passive [2]. Changes in cellular or nuclear mechanics can indicate human diseases, such as inflammation, cardiovascular diseases, infectious diseases, aging, and, most importantly, cancer, as one of the leading causes of death worldwide [13,44,99,100,101].

It has been reported that the detected values of cell mechanical properties, such as viscosity and elasticity, may vary considerably depending on the method used [2,12,26,27,80]. In single-cell studies, such as atomic force microscopy, magnetic twisting cytometry, particle tracking micro-rheology, optical and magnetic tweezers, micropipette aspiration, and cell poking, it is cumbersome to quantify average viscoelastic properties of cells due to the repetition of the experiment to obtain reliable data. Although several single cell studies, including cytometric methods and microfluidic systems, provide high through-put quantitative measurement of mechanical properties, their use is limited due to complex chip design and operational control. Culturing matrix-based differences in mechanical cell properties show limitations in case of microfluidic devices, for instance, for drug screening or in clinical studies [27,59,60]. Atomic force microscopy provides rich viscoelastic properties. Depending on the probe size, cell structures with significantly different responses can be detected. However, discrepancies in the atomic force microscopy could arise due to cantilever calibration errors of 10–15%, speed of indentation, type of probe used, the probed area, and probed timescale [2]. Similarly, each technique is developed under the trade-off relationship between information content and the throughput [96,102].

Quantifying the impact of drugs on the mechanical cell properties can help in the pharmacological screening, for instance, of new components to screen their efficiency, minimize side effects, and to overcome drug resistance [7,103]. To this end, the detection of their influence on the linear viscoelastic cell properties needs to be established. To unravel this regime, a reliable quantitative determination of the average rheological quantities is necessary. The recently modified narrow-gap rotational rheometer provides a promising tool to quantitatively determine those average linear viscoelastic cell properties in single experimental runs [20]. In this technique, cells are adhered to both plates by an adhesive protein, such as fibronectin. The elastic modulus measured with this technique is comparable to atomic force microscopy using a sharp conical probe [2]. In cell monolayer rheology, with the increasing stress, the tensile pre-stress changes the apparent elastic modulus of cells [2,83]. These findings are comparable to the typical tensile stresses measured in single cell studies [2].

The choice of a cell-based mechanical method depends critically on the biological material and the specific question addressed. Similar to other single cell-based mechanical techniques, the recently developed narrow-gap device can also evaluate samples in in vitro and ex vivo settings and can provide reliable data by measuring the average mechanical properties of cell monolayers. By measuring the characteristic response of cells to the drugs, efficient and accurate results can be obtained in a wide variety of areas, such as the quantification of chemical agents and measuring the impact of new drug candidates.

## Figures and Tables

**Table 1:** Comparison of common methods for measuring cell mechanical properties.

Method	Experimental Condition					Measured Moduli	Advantages	Limitations	Reference
	Tissue	Single Cell	Adherent Cells	Suspended Cells	Perturbations in Real Time				
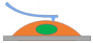 Atomic force microscopy	x	x	x	-	x	E	- Easy sample preparation - High spatial resolution: up to 10 nm - Allows quantitative measurement of shear modulus - Wide range of forces: up to 100 nN - Provides information about depth-dependent mechanical properties at different regions of the cell - Commonly used method for high resolution mechanical measurements	- Low throughput - Can damage cell membrane during deformation - Limited vertical range and magnitude	[2,29,30,31,32]
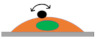 Magnetic twisting cytometry	x	x	x	-	x	G	- Wide frequency range: 0.01~1000 Hz - Allows measurement of more than 100 cells in parallel - Allows monitoring deformation by applying a local force at different regions of the cell - Allows magnitude and frequency control of the applied force	- Limited specific torque of less than 140 Pa	[33,34]
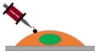 Magnetic tweezers	x	x	x	-	x	G	- Simple and robust experimental configuration - Wide range of forces: up to 100 nN - Good performance measurement: up to 30 cells/h - Allows parallel measurement of multiple cells	- Used for unidirectional forces only - Frequency modulation not possible	[6,25,35,36,37]
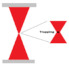 Optical tweezers	x	x	x	-	x	G	- High time resolution of stiffness charges: less than 1 s - Good performance measurement: up to 30 cells/h - Accurate force resolution - Can be used for liquid medium environment	- Limited forces of less than 500 pN - Heating due to laser traps - Imprecise trapping of small particles - Requires calibration before each experiment	[38,39,40]
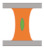 Microplate rheometer	x	x	x	-	x	E	- Wide range of forces: up to 1 N - Possible to control cellular pre-stress	- Low throughput - No subcellular resolution	[41,42,43,44]
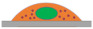 Particle-tracking microrheology/ Nanorheology	x	x	x	-	x	G	- Possible to quantitatively measure the shear modulus - Possible to use under physiological conditions - Good for materials with complex microenvironments - High frequency range: up to 100 kHz	- Used only for soft materials: G <100 Pa - Requires large amount of data to achieve statistical accuracy - Measures the viscoelastic properties of cytoplasm only not of nucleus	[45,46,47]
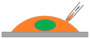 Micropipette aspiration	x	x	x	-	x	E	- Wide range of forces: up to pN - Easy and low-cost setup - Highly accurate measurement of non-linear deformation - Can be used for soft and rigid cells	- Special resolution is limited to a few microns - Low throughput - Can cause cell damage during deformation - Theoretical models-dependent quantitative measurements - Pipette geometry-limited measurements	[48,49,50,51]
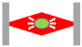 Optical stretching	-	x	-	x	x	E	- High measurement throughput: >100 cells/h - Non-invasive and non-destructive method - Multiple direction probe - No direct physical contact required for mechanical measurements of cells - Simple setup requiring less time for measurements	- Requires extensive modeling for force profile - Temperature can affect sample due to laser-induced heating - Only used for cells in suspension	[38,52,53,54,55]
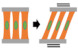 Cell monolayer rheology	x	-	x	-	x	G	- Direct measurement of average mechanical properties of up to 10^6^ cells in a single experimental runs - High reproducibility and easy to perform - Allows measuring both linear and non-linear viscoelastic properties - Allows controlling a wide range of criteria, such as frequency, amplitude, time, and force during a rheological measurement - Allows measuring adhesion limit of tissues or cells in a monolayer	- Results depend on the gap between the plates	[11,20,56,57,58]
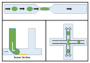 Microfluidic techniques	-	x	x	x	x	G	- Ease of automation - Reduced reagent consumption - Device design and experimental flexibility - Robust, high throughput measurement of cell deformability - Can be used for homogenous and heterogeneous cell populations	- Additional image-based processing may be needed to evaluate cell deformation - Complex chip design and operational control - Results could differ based on the properties of culturing surface - Challenging to use sample in small volumes	[27,59,60,61]

Based on works from Kollmannsberger and Fabry [6] and Wu et al. [2] and expanded; E: Young’s modulus; G: Shear modulus.

**Table 2:** Applications of narrow-gap rheology for cell mechanical studies.

Type of Cells	Measured Moduli	Gap Width (µm)	Gap-Width Precision (µm)	Characteristic Frequency	Characteristic Features	Reference
**3T3 fibroblasts**	G′ and G″	10	±1	Amplitude sweep: 5 Hz Frequency sweep: 0.1–10 Hz	- Allows studying of about 10^6^ cells in a single measurement - Possibility of frequency, amplitude, time, and force-controlled measurements - Allows measuring cell adhesion strength - Allows studying the mechanical properties of cell monolayer or tissues	[56]
**Stromal vascular cells**	G_r_	5	±0.25	-	- Linear cell monolayer rheometer allows analysis of cell mechanical properties by shearing an entire cell monolayer - Allows step-strain experiments - Allows studying cell mechanics of adherent cells with simultaneous live cell imaging of cell deformation	[67]
**3T3 fibroblasts**	Adhesion strength and G*	6.89	-	-	- Allows quantifying mean value of about 106 cells in a single measurement - Allows measuring cell adhesion strength - Uses plate–ring geometry to minimize the differences in shear as a function of radial distance - Uses microbeads to adjust the gap width	[57]
**3T6 fibroblasts ^1^**, **Human fibroblasts**, **HeLa cells ^2^**	G′ and G″	^1^ 5 ^2^ 14	±0.7	Amplitude sweep: 1 Hz Frequency sweep: 0.1–10 Hz	- Direct assessment of a mean value of about 106 cells in a single measurement - No need for treating cells in the rheometer envisions as a fast diagnostic tool - High cell viability in the rheometer - High reproducibility - Allows quantitative measurement of the impact of pre-stress on G′ and G″ - Possibility of frequency, amplitude, time, and force-controlled measurements - Could be used as a diagnostic tool to study the variation in the rheological cell behavior, such as in transgenic cell lines - Allows measuring cell adhesion and load limits - Allows studying the mechanical properties of cell monolayer or tissues	[92]
**3T6 fibroblasts**	Adhesion limit	40	±1	-	- Enables critical shear stress estimation in low-viscous environments, such as cell culture medium - Fibronectin coating showed strong increase in cell adhesion - Allows shear rates up to 105 s^−1^ - Could be used to characterize cell vitality in terms of their fibronectin production rate	[89]
**MCF-7**	G′ and G″	15	-	Amplitude sweep: 0.5 Hz Frequency sweep: 0.1–10 Hz	- Direct assessment of a mean value of 106 cells in a single measurement - High reproducibility - Possibility of frequency, amplitude, time, and force-controlled measurements - Allows measuring cell adhesion - Allows studying the mechanical properties of dense cell monolayer or tissues - Showed measured elasticity close to the atomic force microscopy with conical probe in the same study	[2]
**Murine 3T6 fibroblasts**	G′ and G″	5	±1	Amplitude sweep: 1 Hz Frequency sweep: 0.1–10 Hz	- Enables studying load limit and adhesion of cells in low viscous conditions, such as cell culture medium, which may have an impact on the cell metabolism - Allows quantifying the impact of different cytoskeleton-affecting chemotherapeutic agents on the storage and loss moduli and on the frequency response	[91]
**MDCK-II epithelial cells**	Adhesion strength, G′ and G″	160–200	-	Amplitude sweep: 1, 5 and 10 rad/s Frequency sweep: 1–100 rad/s	- Direct assessment of a bulk of cells - Allows studying the effect of serum starvation on average rheological properties of cell monolayer - Allows measuring cell adhesion strength	[58]
**MCF-7**	G′ and G″	13	±0.4	Amplitude sweep: 1 rad/s Frequency sweep: 0.1–30 rad/s	- Direct assessment of a mean value of 106 cells in a single measurement - No need for treating cells in the rheometer, envisions as a fast diagnostic tool - High reproducibility - Allows quantitative measurement of the impact of pre-stress on G′ and G″ - Allows measurement of quantitative rheological properties in single experimental runs - Higher cell viability in the rheometer - High precision and data reliability - Possibility of frequency, amplitude, time, and force-controlled measurements - Allows measuring cell adhesion and load limits - Allows studying the mechanical properties of cell monolayer or tissues - Allows live quantification of cell strain values during deformation	[93]

G′: Storage modulus; G″: Loss modulus; G_r_: Relaxation modulus; G*: Dynamic shear modulus.
